# M2 macrophage-derived exosomal miR-193b-3p promotes progression and glutamine uptake of pancreatic cancer by targeting TRIM62

**DOI:** 10.1186/s13062-023-00356-y

**Published:** 2023-01-11

**Authors:** Ke Zhang, Yu-Jie Li, Lin-Jia Peng, Hui-Feng Gao, Lu-Ming Liu, Hao Chen

**Affiliations:** 1 Department of Integrative Oncology, Fudan University Shanghai Cancer Center, No.270 DongAn Road, Shanghai, 200032 China; 2 Department of Oncology, Shanghai Medical College, Fudan University, Shanghai, 200032 China

**Keywords:** Pancreatic cancer, Macrophage, Exosome, MiRNA, Glutamine metabolism

## Abstract

**Background:**

Pancreatic cancer (PC) is a highly lethal malignancy that requires effective novel therapies. M2 macrophages are abundant in the PC microenvironment and promote cancer progression. Exosomes are emerging mediators of the crosstalk between cancer cells and the microenvironment. This study was conducted to explore the role of M2 macrophage-derived exosomes in PC.

**Methods:**

Exosomes derived from M2 macrophages were extracted. miR-193b-3p and TRIM62 were overexpressed or silenced to examine their function in PC. Luminescence assays were used to investigate the interaction between miR-193b-3p and TRIM62. Cell proliferation was examined by EdU staining. Would healing and transwell assays were applied to evaluate cell migration and invasion. Co-immunoprecipitation was used to assess the interaction between TRIM62 and c-Myc. Gene and protein expressions were analyzed by quantitative RT-PCR and immunoblotting, respectively.

**Results:**

M2 macrophage-derived exosomal miR-193b-3p promoted the proliferation, migration, invasion, and glutamine uptake of SW1990 cells. Mechanism study revealed that TRIM62 is a target of miR-193b-3p. TRIM62 inhibited the proliferation, migration, invasion, and glutamine uptake of SW1990 cells by promoting c-Myc ubiquitination. Our data also suggested that TRIM62 expression negatively correlated with miR-193b-3p and c-Myc expression. High-expression of miR-193b-3p and c-Myc predicts poor prognosis, whereas low-expression of TRIM62 predicts poor prognosis in patients with PC.

**Conclusion:**

M2 macrophage-derived exosomal miR-193b-3p enhances the proliferation, migration, invasion, and glutamine uptake of PC cells by targeting TRIM62, resulting in the decrease of c-Myc ubiquitination. This study not only reveals the mechanism underlying the crosstalk between M2 macrophages and PC cells but also suggests a promising therapeutic target for PC.

**Supplementary Information:**

The online version contains supplementary material available at 10.1186/s13062-023-00356-y.

## Introduction

Pancreatic cancer (PC) is the seventh cause of cancer-related deaths worldwide [1]. With a 5-year survival rate of 10%, PC represents an increasing cause of cancer-related deaths [2]. Early diagnosis of PC is difficult. It often presents at an advanced stage with highly invasive tumor cells [3]. Risk factors include nonmodifiable (age, sex, genetic susceptibility, etc.) and modifiable (smoking, alcohol, obesity, etc.) [3]. Despite the development of cancer treatments, the incidence of PC is increasing, especially in the western world [1], which may be due to the extensive tumor microenvironment (TME) in PC. In this context, the dysregulation of metabolic reprogramming and immune regulation plays a vital role in the TME [4, 5] and closely associates with progression and treatment efficacy in cancer. Therefore, it is important to elucidate the relationship between tumor cells and the tumor immune–metabolism microenvironment. Tumor-suppressive macrophages (M1 type) and tumor-supportive macrophages (M2 type) constitute the major cell types of the tumor microenvironment, and M2 macrophage-associated markers are primarily associated with a poor clinical outcome [6]. It is well known that M2 macrophages act as a driving factor in tumor-associated macrophages, which generally promote tumor growth, malignance, and metastasis [7].

Exosomes are extracellular vesicles (30–150 nm) implicated in regulating different biological processes [8]. They can be produced by most cells, including macrophages [9]. Macrophage-derived exsosomes can deliver bioactive molecules to target cells to affect their function. Studies have demonstrated that M2 macrophage-derived exosomes facilitate T-cell exhaustion in liver cancer [10], enhance invasion in colon cancer, and regulate BRG1 expression in response to TME [11]. MicroRNAs (miRNAs) are small noncoding RNAs (∼22 nt) that regulate gene expression [12]. Studies have shown that miRNAs can be contained by exosomes [13, 14]. Macrophage exosomal miRNAs have also been reported to affect tumorigenesis. For instance, M1 macrophage-derived exosomal miR-628-5p inhibits the development of hepatocellular carcinoma [15], whereas M2 macrophage-derived exosomal miR-3917 promotes the progression of lung cancer [16]. M2 macrophage-derived exosomal miR-21-5p, miR-155-5p, miR-221-5p, and miR-501-3p promote the differentiation and activity of PC stem cells [17] and the growth, angiogenesis [18], migration, and invasion of PC cells [19]. Furthermore, miR-193b-3p expression is upregulated in plasma exosomes obtained from patients with PC [20], and exosomal miR-193b-3p regulates the chemosensitivity of seminoma to cisplatin through ZBTB7A signaling [21]. Nevertheless, there is no information on bioactive transmission by macrophage-derived exosomal miR-193b-3p to tumor cells in PC.

Tripartite motif (TRIM)-containing proteins are defined by the presence of an N-terminal RING finger, one or two B-boxes, and a coiled-coil domain [22]. Due to the N-terminal RING finger domain, which predominantly contributes to E3 ubiquitin ligase activity, TRIM proteins have been implicated in ubiquitination and are proposed to be a subfamily of E3 ligases. TRIM-containing proteins participate in various cellular processes, such as oncogenesis. For example, TRIM15 promotes the invasion and metastasis of PC cells by mediating APOA1 ubiquitination and degradation [23]. TRIM50 suppresses PC progression and reverses the epithelial–mesenchymal transition by facilitating the ubiquitous degradation of Snail1 [24]. In addition, loss of TRIM29 suppresses the cancer stem cell-like characteristics of PC by accelerating the degradation of ISG15 [25]. Another TRIM member, TRIM62, has been demonstrated to regulate antifungal immunity and intestinal inflammation by facilitating CARD9 ubiquitination [26]. TRIM62 also promotes the proliferation and invasion and increases the chemosensitivity of hepatocellular carcinoma cells by affecting the NF-κB pathway [27] and suppresses tumor proliferation and metastasis in cervical cancer through c-Jun/Slug signaling [28]. Despite the development of scientific research, the role of exosomal miR-193b-3p/TRIM62 in PC cells remains to be elucidated. In this study, we investigated the mechanism by which exosomal miR-193b-3p derived from M2 macrophages participates in the proliferation, migration, invasion, and glutamine uptake of PC cells.

## Materials and methods

### Cell lines and cell culture

SW1990 cells were obtained from ATCC (USA). Cell lines were cultured in DMEM containing 1% penicillin/streptomycin and 10% fetal bovine serum (FBS; Gibco, Grand Island, NY, USA) at 37 °C with 5% CO_2_ humidification.

### Cell transfection

miR-193b-3p mimic (5ʹ-AACUGGCCCUCAAAGUCCCGCU-3ʹ), miR-193b-3p inhibitor (5ʹ-AGCGGGACUUUGAGGGCCAGUU-3ʹ), and negative control (NC, 5ʹ-CACUACUAUUGUGUAGAACAC-3ʹ) were purchased from Beyotime (Suzhou). The control pcDNA3.1 plasmid, TRIM62 overexpression plasmid, and c-Myc overexpression plasmid (Clontech, USA) were generated by Generay Technologies (Shanghai, China). Small interfering RNA (siRNA) targeting TRIM62 (sense, 5ʹ-GCAAGCUCUGCUCUUACUUTT-3ʹ; antisense 5ʹ-AAGUAAGAGCAGAGCUUGCTT-3ʹ) was obtained from Shanghai GenePharma Co., Ltd. Transfection was performed using Lipo2000 (Invitrogen, USA). Empty vector and nonspecific siRNA (siNC) were used as negative controls.

### Adenovirus construction

The control pShuttle-CMV adenovirus, TRIM62 overexpression adenovirus, and c-Myc overexpression adenovirus were obtained from Obio (Shanghai). Transfection was performed using Lipo2000 (Invitrogen, USA) according to the manufacturer’s instructions. At 48 h after transfection, the cell culture medium containing the viral particles was collected to infect SW1990 cells.

### Macrophage polarization and cell co-culture

THP-1 cells were cultured in RPMI 1640 medium supplemented with 10% exosome-free FBS. The medium was changed every 48 h. THP-1 cells were induced to differentiate into M0 macrophages by treatment with 100 ng/mL of phorbol myristic acetate (PMA, Sigma) for 24 h. To induce the M2 polarization of macrophages, 30 ng/mL of IL-4 (R&D Systems Inc., USA) was used to treat M0 macrophages for 24 h.

The conditioned medium (CM) of M0 or M2 macrophages was used for co-culture. To exclude the interference of exosomes in FBS, 10% (*v*/*v*) exosome-free FBS (SunBio, China) was used to configure the medium for the culture of macrophages. After 48 h, the supernatant of macrophages was collected and centrifuged at 5000 rpm for 5 min to remove excess cells. Finally, it was mixed at 1:1 with a complete medium containing 10% (*v*/*v*) exosome-free FBS and added to SW1990 cells for 24 h as the CM. Moreover, GW4869 (Umibio, China) was used as an exosome inhibitor, and macrophages were pretreated with 20 µM GW4869 for 24 h to inhibit the generation and secretion of exosomes.

### Exosome isolation

Briefly, the supernatant medium from the macrophage culture was centrifuged at 500*g* for 15 min, 3000*g* for 15 min, and 12,000*g* for 30 min at 4 h. Exosomes were centrifuged at 140,000*g* for 80 min. After resuspension, they were centrifuged at 140,000*g* for 80 min. Then, the exosomes were placed on the copper grid for examination by transmission electron microscopy (TEM). The exosomes were labeled with a PKH-67 kit (Sigma, St. Louis, MO, USA) to detect their phagocytosis by SW1990 cells. SW1990 cells were treated with 100 µg/mL of M0 or M2 macrophage-derived exosomes.

### EdU assay

Cell proliferation was determined using EdU assays. After the abovementioned treatment, cells were seeded into 24-well plates, incubated with fresh media containing 50 nM EdU reagent (RiboBioInc, China) for 2 h at 37 °C, fixed with 4% paraformaldehyde solution, followed by DAPI staining, and then subjected to fluorescence microscopy (Olympus, Tokyo, Japan).

### Wound healing assay

The migration abilities of SW1990 cells were evaluated using wound healing assays. After the abovementioned treatment, SW1990 cells were seeded in a 35-mm culture dish (8 × 10^5^ cells/dish). Wounds were created using a sterile pipette tip when the cells were fully confluent. After washing with phosphate-buffered saline (PBS) to clean the exfoliated cells, the cells were cultured in a pure medium without FBS. The images of wounds at the same position were taken at 0, 24, and 48 h to calculate the distance.

### Transwell invasion assay

After the abovementioned treatment, transwell chambers were used to examine cell invasion. A culture medium containing 10% FBS was added to the lower chamber. Then, 5 × 10^4^ SW1990 cells were suspended in serum-free culture medium and inoculated in the matrigel-coated upper chamber (BD Biosciences, USA). After 24 h of incubation, the SW1990 cells were removed from the upper chamber with a cotton swab. The cells that penetrated and adhered to the bottom of the filter membrane were fixed with 4% paraformaldehyde in PBS for 10 min, stained with 0.5% crystal violet for 20 min, and then imaged under a microscope.

### Glutamine uptake assay

After the abovementioned treatment, a glutamine assay kit (ab197011; Abcam, USA) was used to determine the concentration of glutamine according to the manufacturer’s protocol. Based on the principle of glutamine conversion into glutamic acid and ammonia, the amount of glutamine was calculated by measuring the amount of ammonia.

### Luciferase activity assay

SW1990 cells were transfected with an miR-193b-3p mimic/inhibitor. Then, the wild-type pGL3-promoter TRIM62 3ʹUTR (WT) or the mutant-type pGL3-promoter TRIM62 3ʹUTR (MUT) luciferase plasmid was transfected into SW1990 cells. The pRL-TK vector was transfected into SW1990 cells, which served as an internal control reporter. A dual-luciferase assay was performed according to the manufacturer’s protocol. The luciferase activity was evaluated using a Dual-Luciferase Reporter Assay system (Promega Biotech Co, Ltd, Beijing, China) at 48 h posttransfection and normalized to Renilla luciferase activity.

### Quantitative RT-PCR (qRT-PCR)

RNAs were isolated using TRIzol and reverse-transcribed using Superscript II (Invitrogen, Shanghai). SYBR master mix (Bio-Rad, Philadelphia, PA) was used for qRT-PCR that was performed using the primers listed in Additional file 1: Table S1. The fold change for the relative gene expression was determined using the 2^−ΔΔCt^ method. *GAPDH*/*U6* served as an internal reference gene.

### Immunoblotting

Cells were washed separately with ice-cold PBS, gently scraped with a cell spatula, and lysed using the radioimmunoprecipitation assay (RIPA) cell lysis buffer (Beyotime, Shanghai, China). Quantification was performed using the BCA protein concentration assay kit (Beyotime, Shanghai, China). Total protein samples were separated by SDS-PAGE and transferred to PVDF membranes. After blocking in 5% nonfat milk, the membranes were probed with antibodies against TRIM62 (ab102012; Abcam), c-Myc (ab32072), TSG101 (ab125011), CD9 (ab92726), CD63 (ab216130), or GAPDH (#5174; CST). The membranes were washed, probed with secondary antibodies (A0208, A0216; Beyotime, Shanghai, China), and then visualized using an ECL kit (Bio-Rad). Results were analyzed using the Image-Pro6.0 software.

### Protein stability

Cycloheximide (CHX, 0.1 mg/ml; Sigma, Shanghai) was used to inhibit protein synthesis. SW1990 cells were treated with CHX for 0, 1, 4, and 8 h, cell lysates were obtained as described earlier, and then protein expression was assayed by western blotting. The CHX treatment time was plotted on the abscissa, and the amount of protein was plotted on the ordinate. The time required to degrade half the protein was the protein half-life.

### Co-immunoprecipitation (Co-IP) and ubiquitination assay

Cell lysates were extracted using a RIPA buffer. The lysates were centrifuged at 12,000×*g* for 10 min at 4 °C. Total cell lysates were used for immunoprecipitation with anti-TRIM62 (ab154635; Abcam), anti-c-Myc (ab168727; Abcam), or normal IgG antibodies (sc-2027; Santa Cruz Biotechnology, Inc.), followed by protein A/G PLUS-Agarose beads (sc-2003; Santa Cruz Biotechnology, Inc.), at 4 °C for 2 h. Immunocomplexes were washed three times in the lysis buffer and subjected to western blotting using anti-TRIM62 (ab102012; Abcam), anti-c-Myc (ab32072; Abcam), or anti-Ubiquitin antibodies (ab7780; Abcam).

### Animal models

Experiments were performed according to the principles of the Committee on Ethics of Animal Experiments of Fudan University Shanghai Cancer Center. In a mice lung metastasis model, 5 × 10^6^ SW1990 cells were inoculated through tail veins (n = 5). Exosomes (10 µg) derived from M0 macrophages (M0-exo) or M2 macrophages transfected with the miR-193b-3p inhibitor (M2/Inhibitor-exo) or NC (M2/NC-exo) were inoculated every 3 days. Otherwise, 5 × 10^6^ SW1990 cells with TRIM62 and c-Myc overexpression adenovirus infection were inoculated through tail veins (n = 5). Mice were euthanized 6 weeks post injection, and the lung was examined and collected for hematoxylin–eosin (HE) staining. The number of lung nodules was recorded.

### Patient samples

Two cohorts of PC cases in Fudan University Shanghai Cancer Center recruited from October 2019 to March 2021 were included in this study. Cohort 1 consisted of patients with tumor and adjacent normal tissues (n = 20), and cohort 2 consisted of patients with tumor (n = 60) and adjacent normal (n = 10) tissues. This study was approved by the Ethics Committee of Fudan University Shanghai Cancer Center and was performed according to the Declaration of Helsinki. Written informed consents were obtained.

### Immunohistochemistry (IHC)

Paraffin-embedded sections of 60 tumor tissues from cohort 2 were prepared for IHC staining. The tissues were fixed with 4% paraformaldehyde, embedded in paraffin, and subjected to standard dewaxing and rehydration. The sections were incubated in citric acid buffer (pH 6.0) for 15 min for antigen retrieval, followed by incubation for 10 min with 3% H_2_O_2_ solution to inactivate endogenous enzymatic activities. Then, the sections were incubated with anti-TRIM62 (ab154635; Abcam) and anti-c-Myc (ab32072; Abcam) antibodies for 1 h at 25 °C, followed by HRP-conjugated anti-IgG antibody for 30 min at 20 °C. After washing three times with PBS, the peroxidase activity was visualized using 3,3ʹ-diaminobenzidine (OriGene Technologies, Inc., Rockville, MD, USA) at 25 °C for 10 s. Then, the sections were counterstained with hematoxylin (OriGene Technologies, Inc.) at room temperature for 3 min. Immunoreactivity was scored using the H-score system by two investigators based on the percentage of positive cells (0–4: 0, < 5%; 1, 5–25%; 2, 25–50%; 3, 50–75%; and 4, > 75%) and staining intensity (0–3: 0, negative; 1, weak; 2, moderate; and 3, strong), which ranged from 0 to 12. Based on the immunoreactivity scores, the patients were categorized into low-expression (H-score < 6) or high-expression (H-score ≥ 6) groups.

### Statistical analysis

Data were analyzed using GraphPad8.4.3 (La Jolla, CA). Results are expressed as mean ± SD. Between-group differences were evaluated using Student’s *t*-test or ANOVA. The Kaplan–Meier method and log-rank tests were used to analyze and compare overall survival. *P* values of < 0.05 were considered to indicate statistical significance.

## Results

### M2 macrophage-derived exosomes enhance the proliferation, migration, invasion, and glutamine uptake of SW1990 cells

**Fig. 1:**
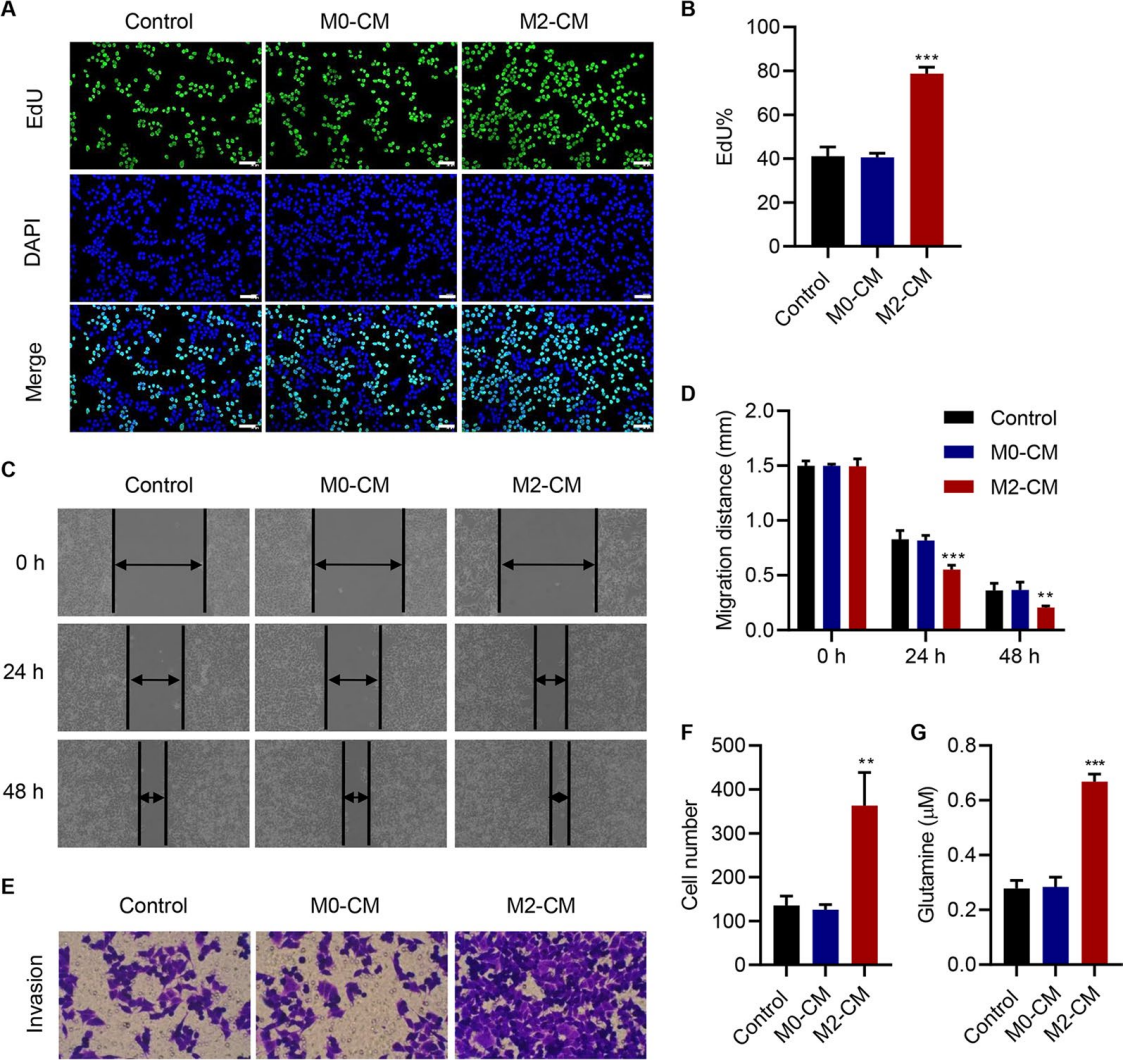
Conditioned medium of M2 macrophages enhances the proliferation, migration, invasion, and glutamine uptake of SW1990 cells. Effect of conditioned medium of M0 macrophages (M0-CM) and M2 macrophages (M2-CM) on **A**, **B** the proliferation, **C**, **D** migration, **E**, **F** invasion, and **G** glutamine uptake of SW1990 cells. Scale bar: 50 μm. Data are expressed as mean ± SD (n = 3). **P < 0.01, ***P < 0.001 versus M0-CM

To explore the effect of macrophages on PC cells, THP-1 cells were treated with PMA and IL-4 to obtain M0 and M2 macrophages, respectively (Additional file 1: Figure S1A), and the expression levels of the macrophage marker CD68 and the M2 macrophage markers CD206 and ARG1 were measured to confirm the differentiation of macrophages (Additional file 1: Figure S1B). Next, we used a co-culture system to mimic the in vivo circumstance and examine the cell–cell communication of macrophages and SW1990 cells. Exosome-free condition medium (CM) was used in the co-culture system of macrophages and SW1990 cells. We found that the CM of M2 macrophages could substantially increase the proliferation (Fig. 1A, B), migration (Fig. 1C, D), invasion (Fig. 1E, F), and glutamine uptake (Fig. 1G) of SW1990 cells compared with the CM of M0 macrophages.

**Fig. 2:**
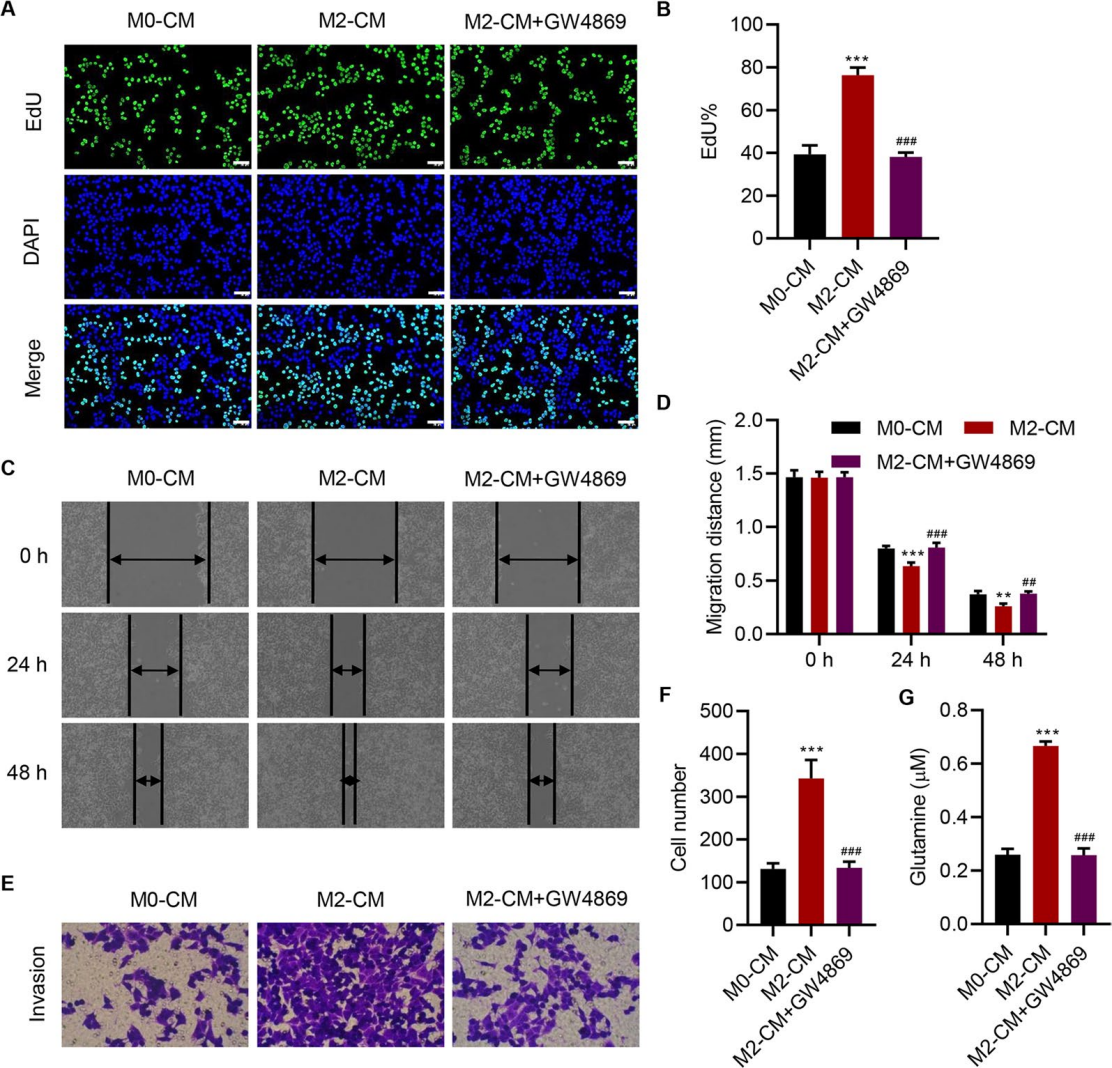
M2 macrophage-derived exosomes enhance the proliferation, migration, invasion, and glutamine uptake of SW1990 cells. Effect of conditioned medium of M0 macrophages (M0-CM) or M2 macrophages treated with GW4869 (M2-CM + GW4869) on the **A**, **B** proliferation, **C**, **D** migration, **E**, **F** invasion, and **G** glutamine uptake of SW1990 cells. Scale bar: 50 μm. Data are expressed as mean ± SD (n = 3). **P < 0.01, ***P < 0.001 versus M0-CM. ^##^*P* < 0.01, ^###^*P* < 0.001 versus M2-CM

To elucidate how the CM of M2 macrophages affects SW1990 cells, the exosomes were successfully isolated, characterized by TEM (Additional file 1: Figure S1C), and confirmed by measuring the expression of exosome markers, including CD9, CD63, and TSG101 (Additional file 1: Figure S1D). The uptake of exosomes by SW1990 cells was confirmed by PHK-67 labeling (Additional file 1: Figure S1E). Then, the CM of M2 macrophages with or without treatment with the exosome inhibitor GW4869 was collected and used to treat SW1990 cells. Results showed that the administration of GW4869 significantly abolished the increase of proliferation (Fig. 2A, B), migration (Fig. 2C, D), invasion (Fig. 2E, F), and glutamine uptake (Fig. 2G) of SW1990 cells. These findings suggest that the exosomes derived from M2 macrophages enhance the proliferation, migration, invasion, and glutamine uptake of SW1990 cells.

### M2 macrophage-derived exosomal miR-193b-3p enhances the proliferation, migration, invasion, and glutamine uptake of SW1990 cells ***in vitro*** and in vivo

**Fig. 3:**
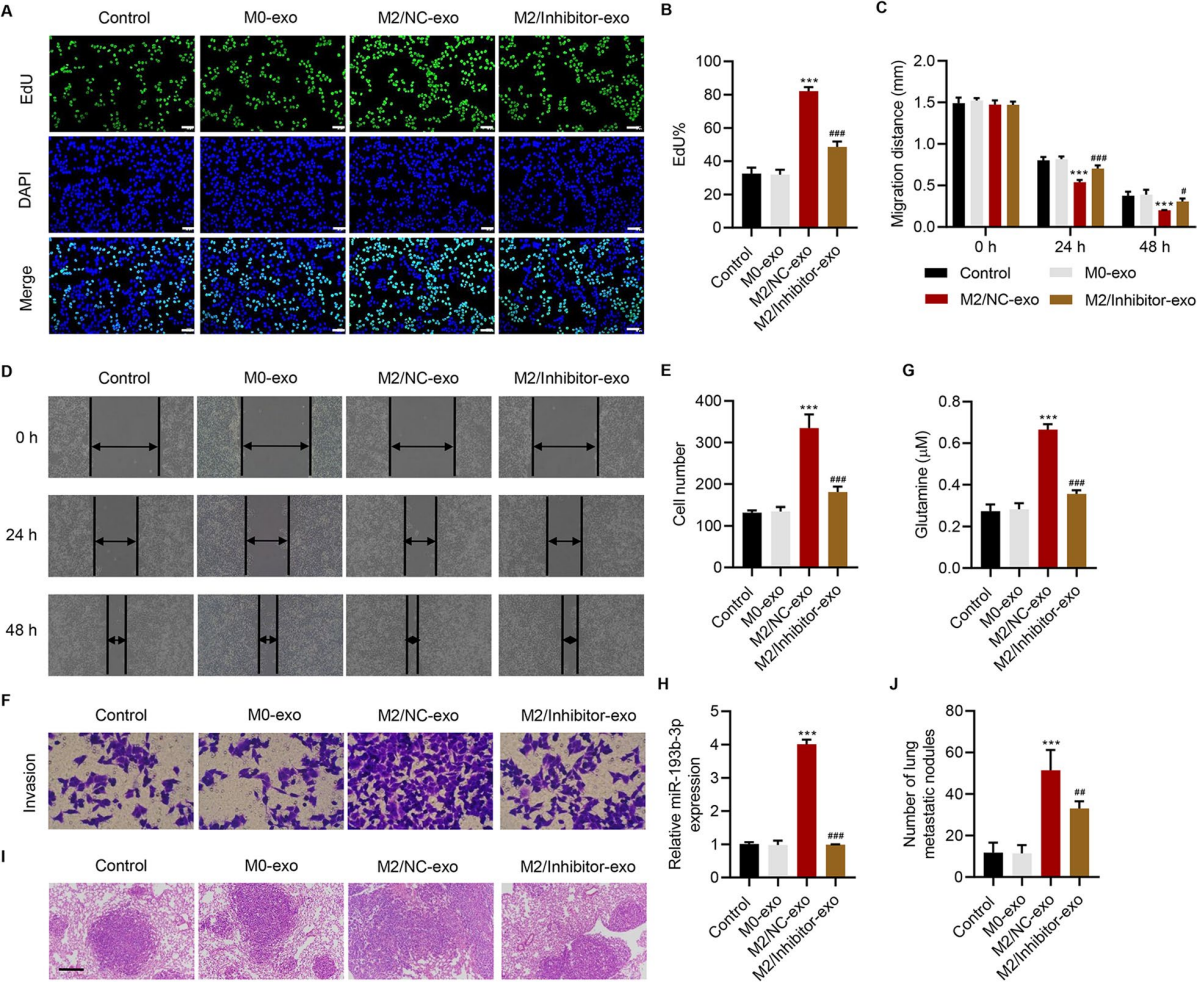
M2 macrophage-derived exosomal miR-193b-3p enhances the proliferation, migration, invasion, and glutamine uptake of SW1990 cells. Effect of exosomes derived from M0 macrophages (M0-exo) or M2 macrophages transfected with the miR-193b-3p inhibitor (M2/Inhibitor-exo) or NC (M2/NC-exo) on the **A**, **B** proliferation (scale bar: 50 μm), **C**, **D** migration, **E**, **F** invasion, **G** glutamine uptake, and **H** miR-193b-3p expression of SW1990 cells (n = 3 per group). Effect of M0-exo, M2/Inhibitor-exo, or M2/NC-exo on **I**, **J** lung metastasis in mice (scale bar: 200 μm) (n = 5 per group). Data are expressed as mean ± SD (n = 3 or 5). ***P < 0.001 versus M0-exo. ^#^*P* < 0.05, ^##^*P* < 0.01, ^###^*P* < 0.001 versus M2/NC-exo.

To further investigate how exosomes derived from M2 macrophages (M2-exo) affect SW1990 cells, we measured the expression of macrophage-exosome-related miRNAs in M0 and M2 macrophage-derived exosomes and observed that the expression levels of miR-193b-3p, miR-502-3p, and miR-222-3p were significantly increased in M2 macrophage-derived exosomes (M2-exo) compared with those in M0 macrophage-derived exosomes (M0-exo) (Additional file 1: Figure S2A). Next, we determined the expression of these three miRNAs in PC tissues obtained from cohort 1 and found that miR-193b-3p showed the most increased expression among the miRNAs; hence, we selected miR-193b-3p for subsequent studies (Additional file 1: Figure S2B-S2D). To examine how miR-193b-3p derived from M2-exo affects SW1990 cells, the miR-193b-3p inhibitor was introduced into M2 macrophages. We found that the exosomes derived from M2 macrophages transfected with the miR-193b-3p negative control (M2/NC-exo) significantly promoted the proliferation (Fig. 3A, B), migration (Fig. 3C, D), invasion (Fig. 3E, F), and glutamine uptake (Fig. 3G) of SW1990 cells, which were inhibited by the exosomes derived from M2 macrophages transfected with the miR-193b-3p inhibitor (M2/Inhibitor-exo). M2/NC-exo also promoted the levels of miR-193b-3p (Fig. 3H) and the numbers of lung metastatic nodules in mice (Fig. 3I, J), which were inhibited by M2/Inhibitor-exo. Overall, these findings suggest that miR-193b-3p derived from M2-exo enhances the proliferation, migration, invasion, and glutamine uptake of SW1990 cells.

### TRIM62 is a target of miR-193b-3p

**Fig. 4:**
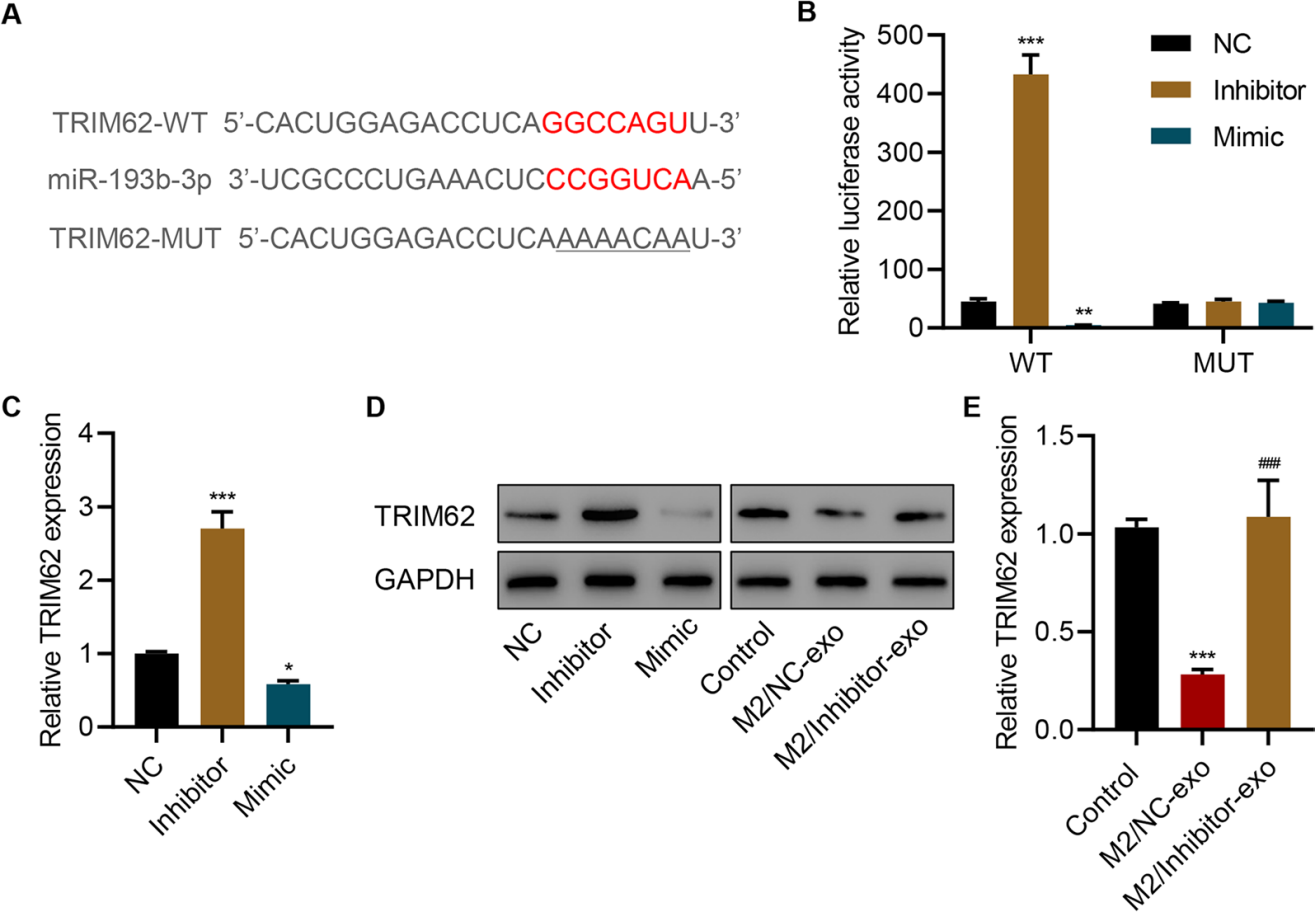
TRIM62 is a target of miR-193b-3p. **A** Sequences of miR-193b-3p and miR-193b-3p-binding site. **B** Effects of miR-193b-3p mimics/inhibitors on the luciferase activity of TRIM62 3ʹUTR wild-type and mutant. Effect of **C**, **D** miR-193b-3p mimics/inhibitors or **D**, **E** M2/Inhibitor-exo or M2/NC-exo transfection on TRIM62 expression in SW1990 cells. Data are expressed as mean ± SD (n = 3). *P < 0.05, **P < 0.01, ***P < 0.001 versus NC or M0-exo. ^###^*P* < 0.001 versus M2/NC-exo.

A bioinformatics analysis was performed to investigate the mechanism underlying the effect of miR-193b-3p. The results indicated a possible binding site of TRIM62 3ʹUTR and miR-193b-3p (Fig. 4A). Next, TRIM62 WT 3ʹUTR or Mut 3ʹUTR and miR-193b-3p inhibitors or mimics were co-transfected. Luminescence assays revealed that inhibiting miR-193b-3p expression strongly upregulated the activity of TRIM62 3ʹUTR, whereas miR-193b-3p mimics strongly downregulated its activity. The miR-193b-3p inhibitors or mimics exerted no effects on the activity of mutant TRIM62 3ʹUTR (Fig. 4B). Results also indicated that the miR-193b-3p inhibitor significantly increased the expression of TRIM62, whereas the miR-193b-3p mimic significantly decreased its expression (Fig. 4C, D). The exosomes derived from M2 macrophages significantly decreased the expression of TRIM62, which was abolished by M2/Inhibitor-exo (Fig. 4D and E). All these findings indicate that TRIM62 is a target of miR-193b-3p.

### TRIM62 regulates miR-193b-3p-mediated proliferation, migration, invasion, and glutamine uptake of SW1990 cells

**Fig. 5:**
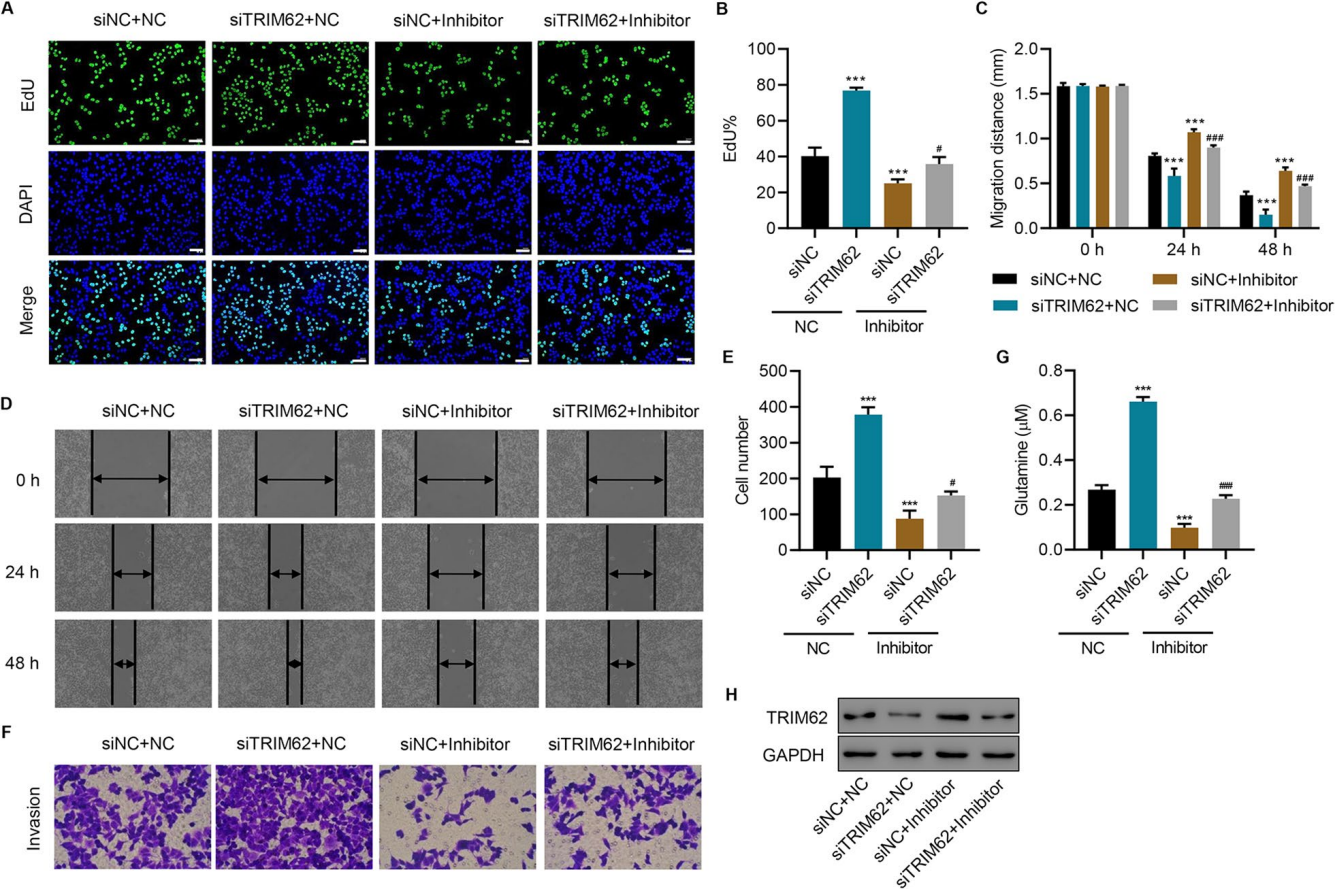
TRIM62 regulates the miR-193b-3p-mediated proliferation, migration, invasion, and glutamine uptake of SW1990 cells. **A**, **B** Cell proliferation, **C**, **D** migration, **E**, **F** invasion, **G** glutamine uptake, and **H** TRIM62 expression of SW1990 cells transfected with miR-193b-3p inhibitor and/or TRIM62 siRNA. Scale bar: 50 μm. Data are expressed as mean ± SD (n = 3). ***P < 0.001 versus siNC + NC. ^#^*P* < 0.05, ^###^*P* < 0.001 versus siNC + Inhibitor.

To further delineate the role of TRIM62, it was successfully silenced in SW1990 cells transfected with the miR-193b-3p inhibitor or NC. TRIM62 silencing significantly promoted the proliferation (Fig. 5A, B), migration (Fig. 5C, D), invasion (Fig. 5E, F), and glutamine uptake (Fig. 5G) of SW1990 cells and decreased the expression of TRIM62 (Fig. 5H). However, the miR-193b-3p inhibitor significantly inhibited the proliferation (Fig. 5A, B), migration (Fig. 5C, D), invasion (Fig. 5E, F), and glutamine uptake (Fig. 5G) of SW1990 cells and increased the expression of TRIM62 (Fig. 5H). All these effects of the miR-193b-3p inhibitor were significantly diminished by TRIM62 silencing (Fig. 5A–H). Meanwhile, TRIM62 was also successfully overexpressed in SW1990 cells treated with M0-exo or M2-exo. Remarkably, TRIM62 overexpression in SW1990 cells significantly ameliorated the M2-exo-induced proliferation (Figures S3A–S3B), migration (Figures S3C–S3D), invasion (Figures S3E–S3F), and glutamine uptake (Figure S3G) of SW1990 cells and the expression of TRIM62 (Figure S3H). Altogether, these findings demonstrate that TRIM62 regulates the miR-193b-3p-mediated proliferation, migration, invasion, and glutamine uptake of SW1990 cells.

### TRIM62 interacts with c-Myc and induces c-Myc ubiquitination

**Fig. 6:**
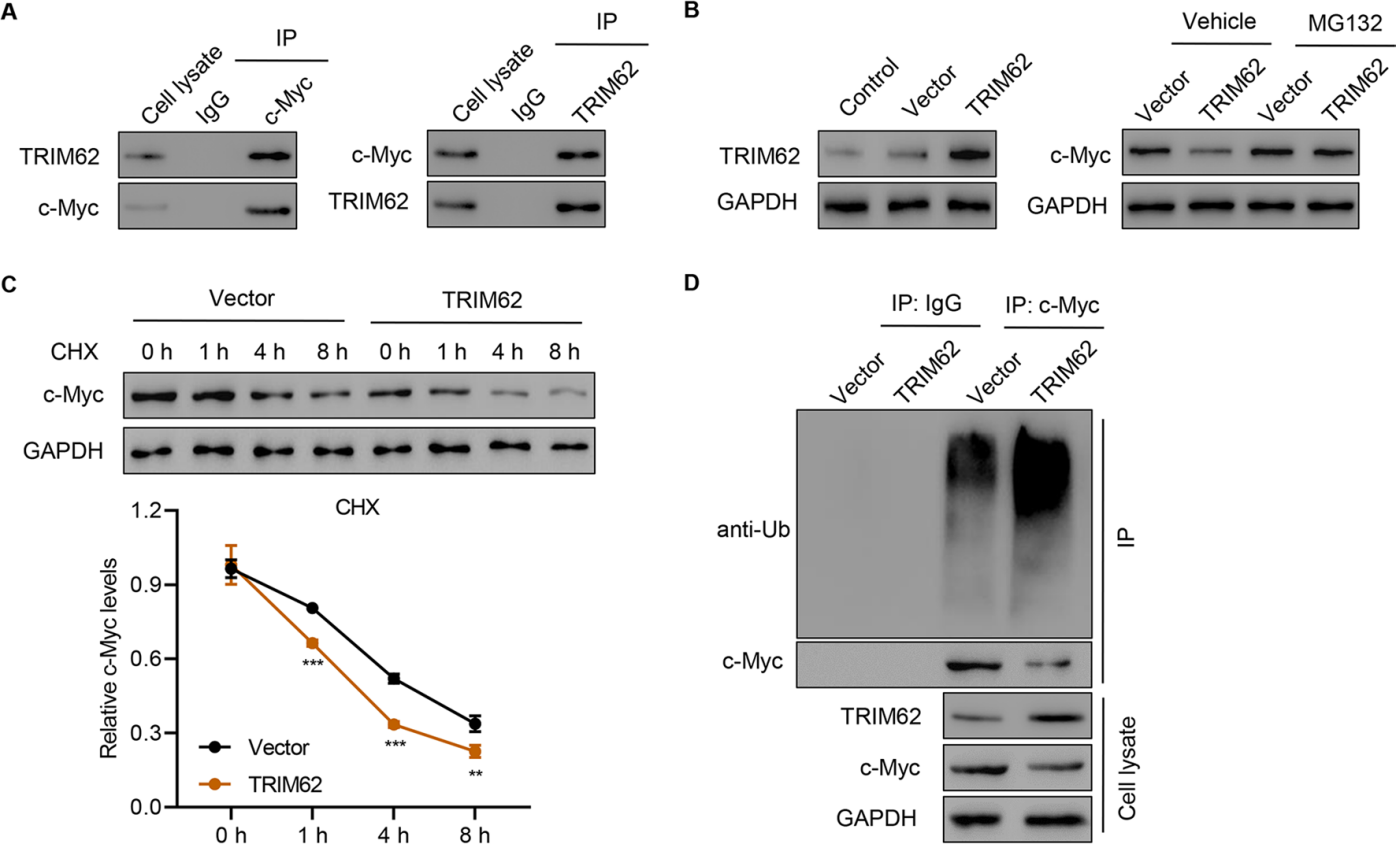
TRIM62 interacts with c-Myc and induces c-Myc ubiquitination. **A** Immunoprecipitation was performed, and immunoprecipitants were probed with indicated antibodies. **B** SW1990 cells transfected with TRIM62 overexpression plasmid were treated with or without MG132 (10 µM), and TRIM62 and c-Myc expressions were evaluated. **C** SW1990 cells transfected with TRIM62 overexpression plasmid were treated with CHX, and c-Myc levels were measured. **D** SW1990 cells transfected with TRIM62 overexpression plasmid were immunoprecipitated with c-Myc for ubiquitination analysis. **P < 0.01, ***P < 0.001 versus vector

To further examine the mechanism by which TRIM62 is involved in migration and invasion, we performed a Co-IP assay using either anti-TRIM62 antibody or anti-c-Myc antibody, which confirmed the interaction between TRIM62 and c-Myc (Fig. 6A). The administration of MG132, a proteasome inhibitor, abolished the TRIM62 overexpression-induced decrease of c-Myc expression (Fig. 6B). TRIM62 overexpression also significantly increased the degradation of c-Myc (Fig. 6C). The IP results also demonstrated that TRIM62 overexpression promoted c-Myc ubiquitination (Fig. 6D). These results indicate that TRIM62 interacted with c-Myc and induced c-Myc ubiquitination.

### C-Myc regulates the TRIM62-mediated proliferation, migration, invasion, and glutamine uptake of SW1990 cells in vitro and in vivo

**Fig. 7:**
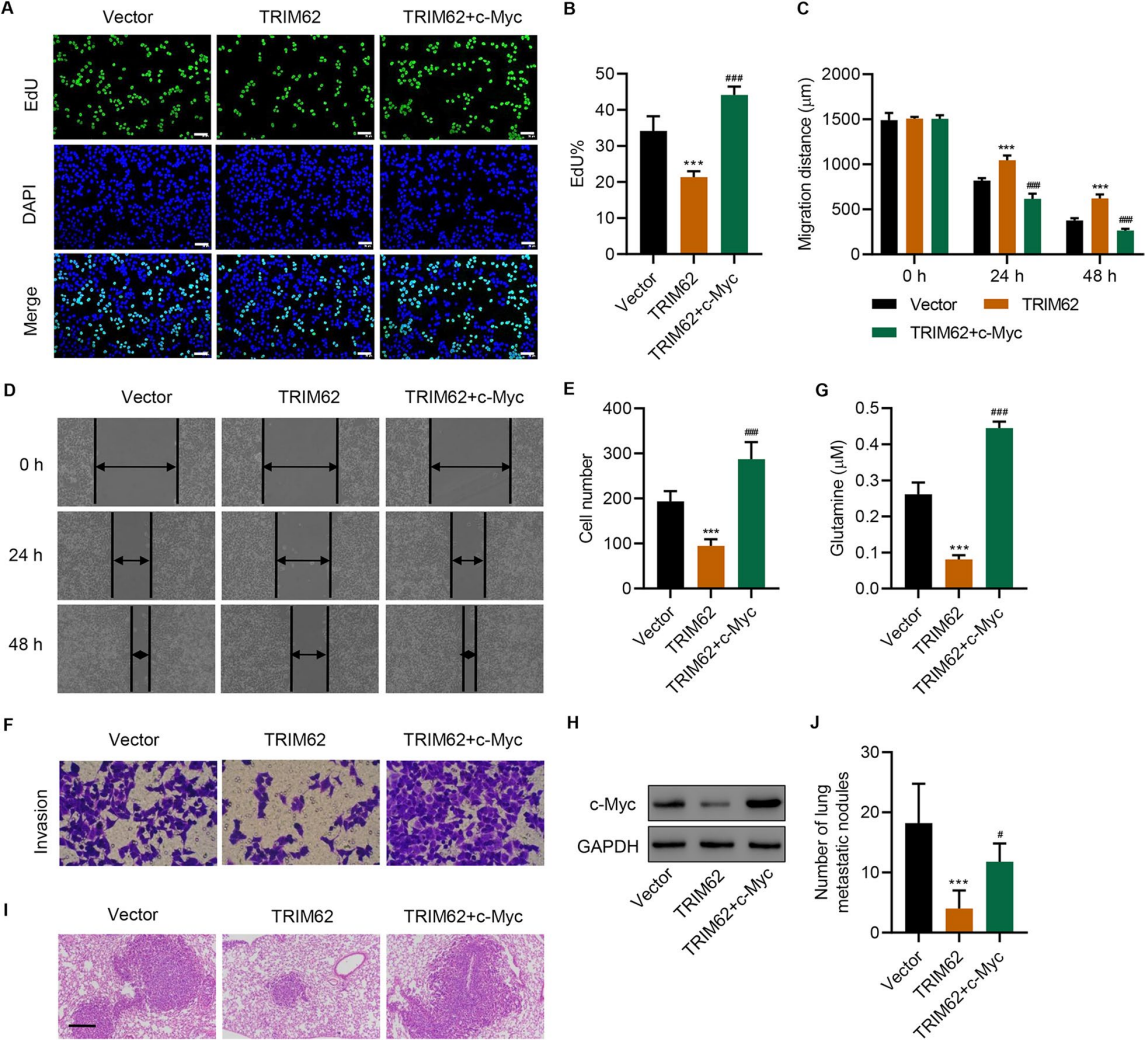
c-Myc regulates the TRIM62-mediated proliferation, migration, invasion, and glutamine uptake of SW1990 cells. **A**, **B** Cell proliferation (scale bar: 50 μm), **C**, **D** migration, **E**, **F** invasion, **G** glutamine uptake, and **H** c-Myc expression of SW1990 cells transfected with TRIM62 overexpression plasmid and c-Myc overexpression plasmid (n = 3 per group). Effect of TRIM62 and c-Myc overexpression on **I**, **J** lung metastasis in mice (scale bar: 200 μm) (n = 5 per group). Data are expressed as mean ± SD (n = 3 or 5). ***P < 0.001 versus vector. ^#^*P* < 0.05, ^###^*P* < 0.001 versus TRIM62

We next examined the effects of c-Myc on proliferation, migration, invasion, and glutamine uptake. Our results showed that c-Myc overexpression significantly reversed the TRIM62-inhibited proliferation (Fig. 7A, B), migration (Fig. 7C, D), invasion (Fig. 7E, F), and glutamine uptake (Fig. 7G) of SW1990 cells. c-Myc expression also reversed the TRIM62 overexpression-induced decrease of c-Myc expression (Fig. 7H) and increased the numbers of lung metastatic nodules (Fig. 7I, J). Overall, these results indicate that c-Myc regulates the TRIM62-mediated proliferation, migration, invasion, and glutamine uptake of SW1990 cells.

### MiR-193b-3p, TRIM62, and c-Myc levels are clinically associated with prognosis of patients with PC

**Fig. 8:**
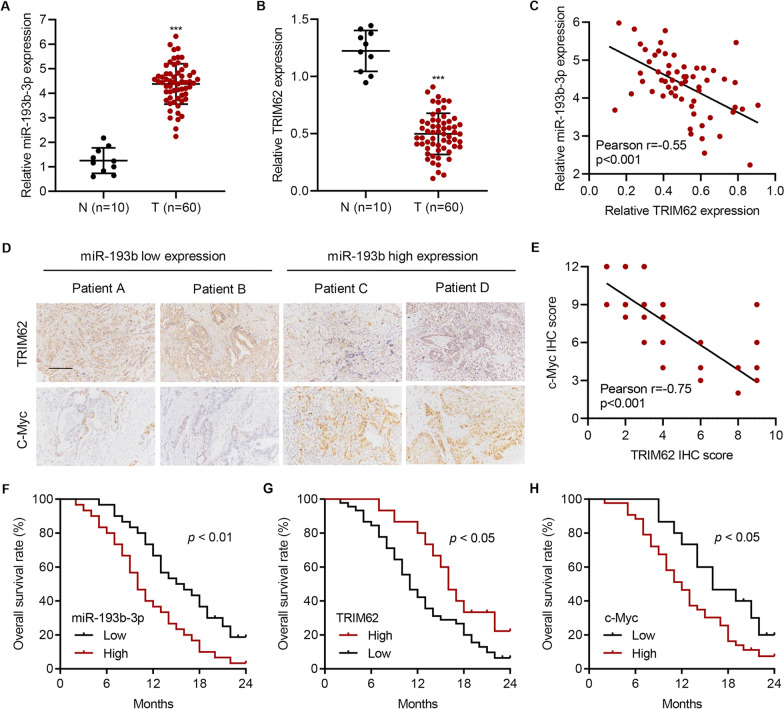
Levels of miR-193b-3p, TRIM62, and c-Myc are clinically relevant in patients with PC. Expression of **A** miR-193b-3p and **B** TRIM62 in PC tissues obtained from cohort 2. **C** Correlation between miR-193b-3p and TRIM62 in cohort 2. **D** IHC staining of TRIM62 and c-Myc in cohort 2. **E** Correlation between TRIM62 and c-Myc IHC scores in cohort 2. Survival was analyzed and compared between patients with high and low levels of **F** miR-193b-3p, **G** TRIM62, and **H** c-Myc expression in tumor tissues obtained from cohort 2. ***P < 0.001 versus N

To analyze the clinical relevance, we measured the levels of miR-193b-3p, TRIM62, and c-Myc in PC tissues obtained from cohort 2. Results showed that compared with than in adjacent normal tissues, miR-193b-3p levels were dramatically increased in PC tissues (Fig. 8A), whereas TRIM62 levels were significantly decreased (Fig. 8B). A correlation analysis suggested a strong negative correlation between TRIM62 and miR-193b-3p (Fig. 8C). We also evaluated the expression of c-Myc and TRIM62 at the protein level by IHC staining (Fig. 8D) and analyzed the correlation. TRIM62 expression negatively correlated with c-Myc expression (Fig. 8E). Analysis of the overall survival rate showed that patients with high-expression of miR-193b-3p had a low survival rate, patients with high-expression of TRIM62 had a high survival rate, and those with high-expression of c-Myc had a low survival rate (Fig. 8F–H). These results suggest that TRIM62 negatively correlates with miR-193b-3p and c-Myc, and high expressions of miR-193b-3p and c-Myc predict poor prognosis, whereas low-expression of TRIM62 predicts poor prognosis.

## Discussion

This study demonstrated that M2 macrophage-secreted exosomal miR-193b-3p enhances the proliferation, migration, invasion, and glutamine uptake of SW1990 cells. The mechanism study indicated that exosomal miR-193b-3p targets TRIM62 that interacts with and induces c-Myc ubiquitination, resulting in the promotion of the proliferation, migration, invasion, and glutamine uptake of SW1990 cells. Our data also indicate that TRIM62 negatively correlates with miR-193b-3p and c-Myc, and high expressions of miR-193b-3p and c-Myc predict poor prognosis, whereas low-expression of TRIM62 predicts poor prognosis. To our knowledge, our study is the first to indicate that miR-193b-3p**/**TRIM62/c-Myc signaling promotes the proliferation, migration, invasion, and glutamine uptake of PC cells.

The TME consists of different types of cells, including cancer cells and macrophages. Tumor-associated macrophages counteract the cytotoxic effects of NK/T cells, promoting cancer proliferation and migration [29]. As the major immune cells in the TME, M2 macrophages exhibit immunosuppressive properties. Increasing evidence suggests that exosomes can transfer cargoes to target cells to promote cancer progression and metastasis [30]. It has been demonstrated that the transmission of HISLA through macrophage-secreted exosomes increases the apoptotic resistance of breast cancer cells [31]. M2-exos enhance cell migration in colon cancer through miRNAs [11]. In this study, we found that M2-exos enhanced the proliferation, migration, invasion, and glutamine uptake of SW1990 cells. Moreover, this effect was mediated by miR-193b-3p**/**TRIM62/c-Myc signaling. These findings improve our understanding of the crosstalk between macrophage-derived exosomes and the TME and also broaden our understanding of PC progression.

miR-193b-3p is involved in different biological and pathological processes. For instance, Li et al. reported that miRNA-193b suppresses proliferation and induces apoptosis in ovarian cancer [32]. miR-193b-3p also inhibits neuroblastoma cell growth [33]. Furthermore, decreasing the expression of miR-193b was found to impair PC cell growth [34]. The present study demonstrated that miR-193b-3p expression was dramatically upregulated in M2-exo, and exosomal miR-193b-3p enhanced the proliferation, migration, invasion, and glutamine uptake of SW1990 cells through TRIM62/c-Myc signaling. These findings indicate a novel role for miR-193b-3p in the progression of PC.

TRIM62 has been implicated in different types of diseases. For example, Cao et al. indicated that TRIM62 promoted CARD9 ubiquitination to promote antifungal immunity and decrease susceptibility to fungal infection [26]. Moreover, TRIM62 was found to be activated in the muscles of critically ill patients [35]. Quintas-Cardama demonstrated that NSCLC lesions lose TRIM62 in a stepwise manner during disease progression [36]. In the present study, we demonstrated that TRIM62 overexpression significantly ameliorated the exosomal miR-193b-3p-promoted proliferation, migration, invasion, and glutamine uptake of SW1990 cells. These data widen our understanding of TRIM62 and PC progression.

Glutamine is a major nutrient that participates in different aspects of cancer metabolism. It supports both biosynthesis and the tricarboxylic acid cycle [37, 38]. A hallmark of metabolism reprogramming in cancer cells is the increased utilization of glutamine [39]. Demas et al. reported that glutamine metabolism drives the growth of mammary cancer [40]. Blockade of glutamine metabolism was found to suppress cancer growth by reprogramming myeloid cells and metabolically reshaping the TME [41]. In the present study, we confirmed that miR-193b-3p derived from M2-exos enhanced the glutamine uptake of SW1990 cells through TRIM62/c-Myc ubiquitination. These results elucidated a new role for miR-193b-3p/TRIM62/c-Myc in the regulation of glutamine uptake by PC cells. However, there are some limitations in our study. For instance, we used only SW1990 cells for these experiments. We intend to use other PC cells for further experiments. A PDX mouse model will provide more relevant data and will be used in a future study. Despite these limitations, we report a novel mechanism underlying the progression of PC induced by M2 macrophage-derived exosomal miR-193b-3p.

In conclusion, M2 macrophage-secreted exosomal miR-193b-3p enhances the proliferation, migration, invasion, and glutamine uptake of SW1990 cells by targeting TRIM62 and inhibiting c-Myc ubiquitination. These findings emphasize the importance of miR-193b-3p/TRIM62/c-Myc signaling in the progression of PC and provide novel insights into therapeutic strategies.

## Supplementary Information

**Additional file 1. Table S1.** Primer sequences used in the study. **Table S2.** Clinicopathological characteristics and follow-up data of 60 patients with pancreatic cancer. **Figure S1.** Macrophage induction and identification. (**A**) Differentiation and induction schedule. (**B**) Identification of M0 and M2 macrophage. Exosome identification by TEM (**C**; scale bar: 100 nm) and Western blotting (**D**). (**E**) SW1990 cell endocytosis of exosomes (scale bar: 50 µm). Data are expressed as mean ± SD (n = 3).***P<0.001 vs. M0. **Figure S2.** The expression of macrophage exosomes-related miRNAs. (**A**) miRNA profiling in M0/M2-exo. Data are expressed as mean ± SD (n = 3). Expression of miR-193b-3p (**B**), miR-502-3p (**C**), and miR-222-3p (**D**) in tumor tissues obtained from cohort 1. ***P<0.001 vs. M0-exo. **Figure S3.** Overexpression of TRIM62 regulates M2-exo-mediated proliferation, migration, invasion and glutamine uptake of SW1990 cells. (**A**, **B**) Proliferation, (**C**, **D**) migration, (**E**, **F**) invasion, (G) glutamine uptake and (**H**) TRIM62 expression of SW1990 cells transfected with TRIM62 plasmids and treated with M0-exo or M2-exo. Scale bar: 50 µm. Data are expressed as mean ± SD (n = 3). ***P<0.001 vs. Vector+M2-exo.

## Data Availability

The data used to support the findings of this study are included within the article.
