# Supplementary material for: M2 macrophage-derived exosomal miR-193b-3p promotes progression and glutamine uptake of pancreatic cancer by targeting TRIM62
Source: Biol Direct. 2023 Jan 11;18:1. doi: 10.1186/s13062-023-00356-y (PMC9832623; doi:10.1186/s13062-023-00356-y)
Supplement: Supplementary file 1 — Additional file 1. Table S1. Primer sequences used in the study. Table S2. Clinicopathological characteristics and follow-up data of 60 patients with pancreatic cancer. Figure S1. Macrophage induction and identification. (A) Differentiation and induction schedule. (B) Identification of M0 and M2 macrophage. Exosome identification by TEM (C; scale bar: 100 nm) and Western blotting (D). (E) SW1990 cell endocytosis of exosomes (scale bar: 50 µm). Data are expressed as mean ± SD (n = 3).***P<0.001 vs. M0. Figure S2. The expression of macrophage exosomes-related miRNAs. (A) miRNA profiling in M0/M2-exo. Data are expressed as mean ± SD (n = 3). Expression of miR-193b-3p (B), miR-502-3p (C), and miR-222-3p (D) in tumor tissues obtained from cohort 1. ***P<0.001 vs. M0-exo. Figure S3. Overexpression of TRIM62 regulates M2-exo-mediated proliferation, migration, invasion and glutamine uptake of SW1990 cells. (A, B) Proliferation, (C, D) migration, (E, F) invasion, (G) glutamine uptake and (H) TRIM62 expression of SW1990 cells transfected with TRIM62 plasmids and treated with M0-exo or M2-exo. Scale bar: 50 µm. Data are expressed as mean ± SD (n = 3). ***P<0.001 vs. Vector+M2-exo. [file 13062_2023_356_MOESM1_ESM.docx]

**Supplementary Tables**

**Table S1. Primer sequences used in the study**

| Gene | Forward/Reverse | Sequence (5’-3’) |
| --- | --- | --- |
| CD68 | Forward | TCCAGCAGAAGGTTGTCTAC |
|  | Reverse | TGATGAGAGGCAGCAAGATG |
| CD9 | Forward | ATAGGCTTTGGAGGGAATC |
|  | Reverse | TACTGTCGCAGGTATCATC |
| ARG1 | Forward | CGTGGGAGGTCTGACATAC |
|  | Reverse | CCAGGGATGGGTTCACTTC |
| TRIM62 | Forward | GGGTGTCTTCCTGGACTATG |
|  | Reverse | TAGATGCGGACGGTGTTG |
| MYC | Forward | CCTTCTTTCCTCCACTCTC |
|  | Reverse | CAAACCCTCTCCCTTTCTC |
| GAPDH | Forward | AATCCCATCACCATCTTC |
|  | Reverse | AGGCTGTTGTCATACTTC |
| miR-125b-5p | Forward | CGCGTCCCTGAGACCCTAAC |
|  | Reverse | AGTGCAGGGTCCGAGGTATT |
| miR-193b-3p | Forward | GCGAACTGGCCCTCAAAGT |
|  | Reverse | AGTGCAGGGTCCGAGGTATT |
| miR-500a-5p | Forward | CGCGTAATCCTTGCTACCTGG |
|  | Reverse | AGTGCAGGGTCCGAGGTATT |
| miR-502-3p | Forward | CGAATGCACCTGGGCAAG |
|  | Reverse | AGTGCAGGGTCCGAGGTATT |
| miR-99a-5p | Forward | GCGAACCCGTAGATCCGAT |
|  | Reverse | AGTGCAGGGTCCGAGGTATT |
| miR-378a-3p | Forward | CGCGACTGGACTTGGAGTCA |
|  | Reverse | AGTGCAGGGTCCGAGGTATT |
| miR-221-3p | Forward | CGCGAGCTACATTGTCTGCTG |
|  | Reverse | AGTGCAGGGTCCGAGGTATT |
| miR-222-3p | Forward | GCGCGAGCTACATCTGGCTA |
|  | Reverse | AGTGCAGGGTCCGAGGTATT |
| miR-511-3p | Forward | GCGCGAATGTGTAGCAAAA |
|  | Reverse | AGTGCAGGGTCCGAGGTATT |
| U6 | Forward | CTCGCTTCGGCAGCACA |
|  | Reverse | AACGCTTCACGAATTTGCGT |

**Table S2.** Clinicopathological characteristics and follow-up data of 60 patients with pancreatic cancer

| Characteristics | miR-193b-3p expression | | *P* value |
| --- | --- | --- | --- |
|  | Low | High |  |
| **Gender** |  |  | 0.100 |
| Male (n=40) | 23 | 17 |  |
| Female (n=20) | 7 | 13 |  |
| **Age (years)** |  |  | 0.070 |
| < 65 (n=31) | 19 | 12 |  |
| ≥ 65 (n=29) | 11 | 18 |  |
| **Tumor size (cm)** |  |  | 0.015 |
| ≤ 3 (n=21) | 15 | 6 |  |
| > 3 (n=39) | 15 | 24 |  |
| **Lymph node metastasis** |  |  | 0.001 |
| No (n=20) | 16 | 4 |  |
| Yes (n=40) | 14 | 26 |  |
| **T stages**  I+II (n=25)  III (n=35) | 17  13 | 8  22 | 0.018 |

Differences between groups were determined by the Chi-square test.

**Supplementary figures**

**
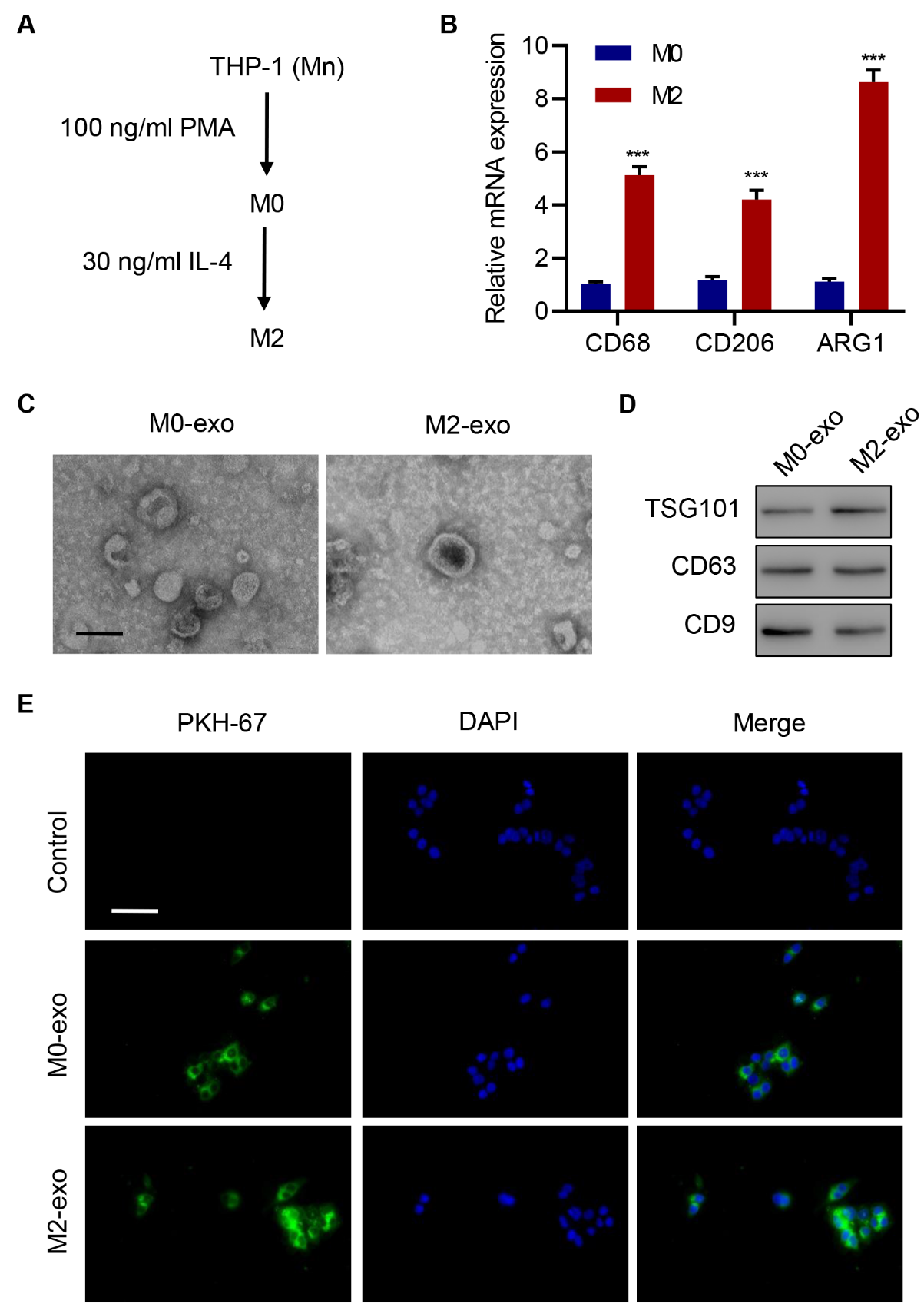
**

**Figure S1. Macrophage induction and identification.** (A) Differentiation and induction schedule. (B) Identification of M0 and M2 macrophage. Exosome identification by TEM (C; scale bar: 100 nm) and Western blotting (D). (E) SW1990 cell endocytosis of exosomes (scale bar: 50 µm). Data are expressed as mean ± SD (n = 3). ****P* < 0.001 vs. M0.

**
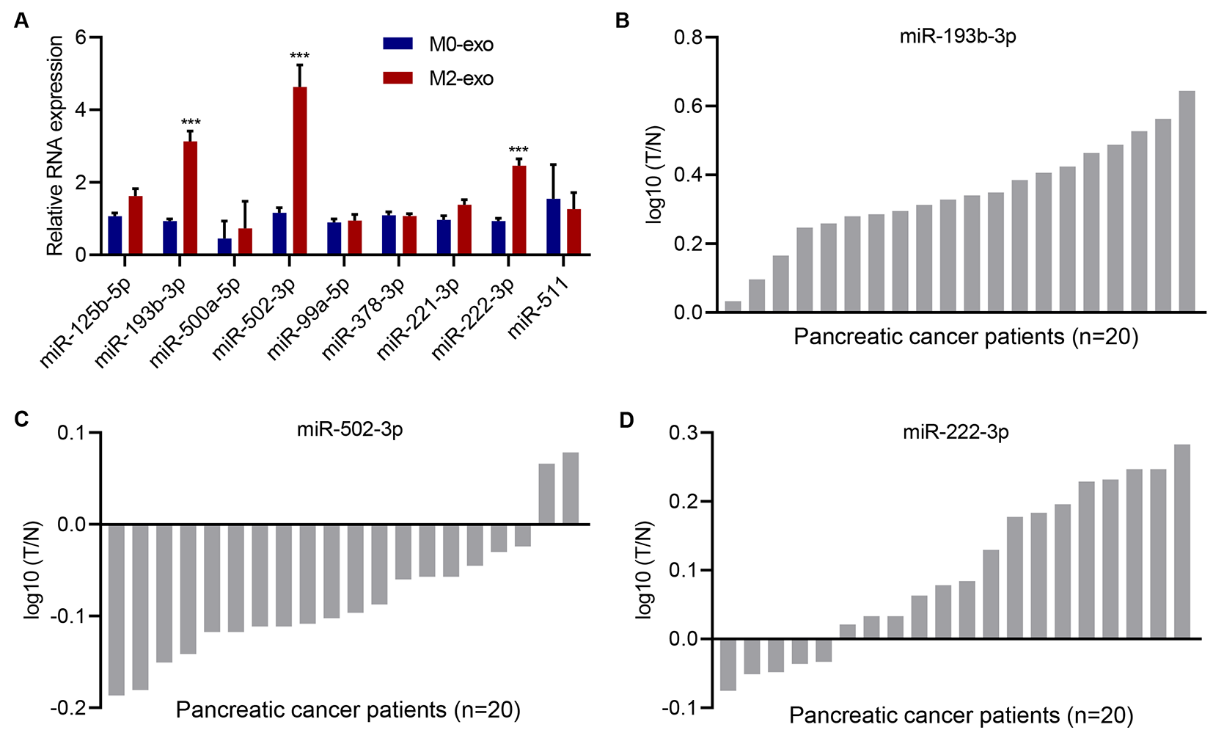
**

**Figure S2. The expression of macrophage exosomes-related miRNAs.** (A) miRNA profiling in M0/M2-exo. Data are expressed as mean ± SD (n = 3). Expression of miR-193b-3p (B), miR-502-3p (C), and miR-222-3p (D) in tumor tissues obtained from cohort 1. ****P* < 0.001 vs. M0-exo.


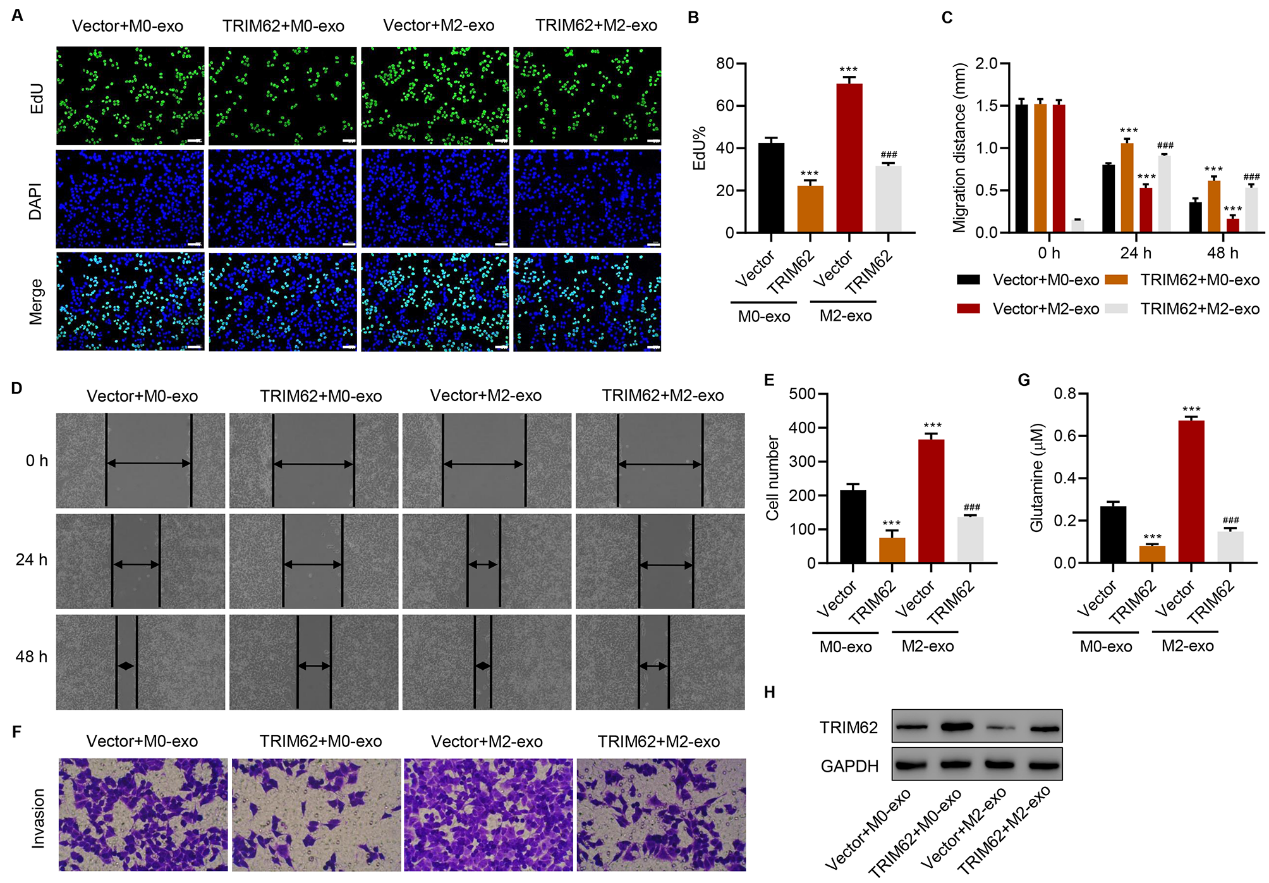


**Figure S3. Overexpression of TRIM62 regulates M2-exo-mediated proliferation, migration, invasion and glutamine uptake of SW1990 cells.** (A, B) Proliferation, (C, D) migration, (E, F) invasion, (G) glutamine uptake and (H) TRIM62 expression of SW1990 cells transfected with TRIM62 plasmids and treated with M0-exo or M2-exo. Scale bar: 50 µm. Data are expressed as mean ± SD (n = 3). ****P* < 0.001 vs Vector+M0-exo. ^###^*P* <0 .001 vs. Vector+M2-exo.
